# Worsening psychosis associated with administrations of buspirone and concerns for intranasal administration: A case report

**DOI:** 10.3389/fpsyt.2023.1129489

**Published:** 2023-02-17

**Authors:** Samuel Apeldoorn, Rebecca Chavez, Freshta Haschemi, Kareem Elsherif, David Weinstein, Tyler Torrico

**Affiliations:** ^1^School of Medicine, Ross University, Miramar, FL, United States; ^2^School of Medicine, American University of the Caribbean, Miramar, FL, United States; ^3^Department of Psychiatry, UCLA-Kern Medical Center, Bakersfield, CA, United States; ^4^American Psychiatric Association, Substance Abuse and Mental Health Services Administration Minority Fellowship, Washington, DC, United States

**Keywords:** adverse reaction, anxiolytic, drug abuse, nasal inhalation, methamphetamine, nasal insufflation, psychopharmacology

## Abstract

Buspirone is commonly used to treat generalized anxiety disorder and demonstrates a limited side-effect profile compared to other anxiolytics. Buspirone is considered generally safe, and neuropsychiatric adverse reactions are uncommon. There are rare clinical case reports that suggest buspirone-induced psychosis. We present a case of buspirone worsening psychosis for a patient psychiatrically hospitalized for an episode of decompensated schizoaffective disorder. The patient had a primary diagnosis of schizoaffective disorder and was treated with antipsychotics during this hospitalization, but his symptoms worsened when buspirone was administered on two separate occasions. During the first trial of buspirone, the patient exhibited traits of increased aggression, odd behaviors, and paranoia. The buspirone was discontinued after the patient admitted to hiding his pills to later consume through nasal ingestion. The second trial resulted in repeated exacerbated symptoms of paranoia related to food and substantially decreased oral intake. Considering its complex mechanism of action, buspirone is suggested to derive its neuropharmacological effects through 5-HT1A receptors. However, the drug also has been found to mediate dopamine neurotransmission. Buspirone acts as an antagonist at presynaptic dopamine D2, D3, and D4 receptors. Yet, contrary to expected outcomes, it was unable to produce antipsychotic effects and instead resulted in a substantial increase in dopaminergic metabolites. The route of administration may also play a role in the enhancement of the buspirone’s effects, particularly considering that after first-pass metabolism, buspirone has approximately 4% oral bioavailability. Intranasal administration of buspirone leads to faster drug absorption by direct transport from the nasal mucosa to the brain and increased bioavailability.

## 1. Introduction

Buspirone is a pharmacologically unique azapirone and orally administered drug approved by the Federal Drug Administration (FDA) in 1986 for the treatment of generalized anxiety disorder. Buspirone was originally developed as an antipsychotic drug but was deemed ineffective and found to be more useful for the treatment of anxiety (1). Buspirone also demonstrates a limited side-effect profile when compared to other anxiolytics (1). Buspirone is considered generally safe, with the most common reported adverse reactions being dizziness (9%), sedation (9%), headache (7%), nausea (6%), fatigue (5%), nervousness (4%), light-headedness (4%), dry mouth (3%) diarrhea (3%), depression (2%), paresthesia (2%), excitation (2%), weakness (2%), and sweating/clamminess (1%) (2, 3). Neuropsychiatric adverse reactions are not common (4–9).

There are very few clinical case reports that suspect orally administered buspirone-inducing psychosis. Buspirone is thought to have a slow onset for its anxiolytic effects. However, Friedman reported a case of the onset of delusions within 72 h after the first administered dose of buspirone that resolved within 48 h after stopping buspirone (4). Similarly, Trachman (5) reported psychosis after a single dose of buspirone 5 mg. Two childhood cases were reported after buspirone administration; one was where a patient developed loose associations, thought blocking, and odd behavior, and the other case was with worsening aggression and bizarre behavior (6). A small case series of seven patients investigated the effects of buspirone in patients with schizophrenia with limited conclusions, but ultimately, it appeared that buspirone had the potential to modify concurrent antipsychotic effects and potentially worsen extrapyramidal symptoms (7). There are also reports of buspirone possibly inducing mania (8, 10), and hypomania (9).

## 2. Case presentation

We present a 33-year-old Black male with schizoaffective disorder – bipolar type, generalized anxiety disorder, stimulant use disorder (methamphetamine type), phencyclidine use disorder, and cannabis use disorder. The patient’s illness at baseline includes residual auditory hallucinations but no suicidal or homicidal ideations. Two months before admission, the patient’s outpatient medication regimen consisted of valproic acid 1000 mg daily, buspirone 15 mg three times a day, trazodone 50 mg daily, gabapentin 900 mg daily, quetiapine 300 mg daily, and paliperidone palmitate 156 mg intramuscular injections every 4 weeks. The patient’s outpatient team reported that the patient frequently nasally ingested his buspirone and had a history of difficulty with medication adherence.

Prior to admission, the patient had abandoned his domicile and 4 days later was found with alcohol intoxication by police and making suicidal statements. The patient was placed on an involuntary psychiatric hold while receiving a medical evaluation. Admission labs were unremarkable except for urine toxicology positive for amphetamines and tetrahydrocannabinol. He was endorsing bizarre and paranoid delusions, such as believing people surrounding him were aliens and demons. Furthermore, he began refusing hospital food, believing that it was poisoned.

On admission to the inpatient psychiatric unit, the patient developed increasing agitation and was offered oral haloperidol 5 mg, lorazepam 2 mg, and diphenhydramine 50 mg. Still, he refused and then later received them *via* intramuscular injections after escalating violence. The patient was offered oral risperidone 2 mg daily to target ongoing psychotic symptoms with diphenhydramine 25 mg nightly; however, the patient refused to accept those medications. He later accepted treatment with oral chlorpromazine 150 mg daily and showed some decrease in agitation.

On the 7th day of hospitalization, the patient requested that his home medication regimen be restarted and complained of uncontrolled anxiety. The patient was initiated on valproic acid 1000 mg daily and buspirone 5 mg three times a day. After 48 h of initiating buspirone, the patient exhibited increased bizarre and paranoid delusions, agitation, homicidal ideations, and disorganized thinking. The patient began refusing his oral medications, claiming he was abducted by aliens and had frequent verbal outbursts toward staff. Chlorpromazine dosage was titrated up to 300 mg daily; however, the patient continued to refuse doses frequently, and his antipsychotic medication was switched to quetiapine XR (extended-release) 300 mg. Valproic acid was also discontinued due to the patient’s inability to cooperate with laboratory monitoring. Quetiapine XR was titrated up to 800 mg and then discontinued due to a lack of therapeutic response. The patient was then started on oral haloperidol and titrated to a dose of 10 mg twice daily. After 8 days, buspirone was discontinued as the patient was found to be hiding buspirone in his mouth and later admitted to reserving it for nasal ingestion. Two days after the discontinuation of buspirone, the patient was calm, in behavioral control, and cooperative with the treatment team.

The patient was on a regimen of haloperidol 30 mg daily and benztropine 4 mg daily while awaiting discharge to a locked psychiatric rehabilitation center. During that time, the patient’s average daily meal intake was 76%, and his medications were switched from haloperidol to a trial of oral Risperdal and, finally, the administration of long-acting injectable paliperidone palmitate 234 mg.

On hospital day 62, the patient insisted on restarting buspirone for anxiety, which was re-initiated. Two days later, the patient’s paranoid delusions about his food being poisoned returned, and his oral intake began to decline significantly. The patient only accepted pre-packaged food during that time, which was a behavioral change compared to the previous. After 4 days, the patient exhibited an increase in aggression. Clinical Global Impressions Severity scale (CGI-S) scored 6 (severely ill) at that time. Buspirone was discontinued on hospital day 72. Over the course of 10 days that the patient received buspirone, his oral intake averaged 32% daily, and he required high amounts of behavioral prompting for feeding. It was noted in behavioral notes during that time that the patient had been found to be nasally ingesting buspirone again. One day after buspirone was discontinued, the patient’s meal intake increased to 83%, with adequate oral intake for the remainder of psychiatric hospitalization. In addition, the patient’s Clinical Global Impressions Improvement scale (CGI-I) scored 2 (much improved). Ultimately, the patient responded well to paliperidone palmitate 234 mg intramuscular every 4 weeks, which was continued throughout the remainder of his hospital course. Buspirone was avoided for the remainder of the hospitalization, and the patient was discharged to a psychiatric rehabilitation center.

## 3. Discussion

The patient described had two separate administration windows of buspirone during this hospitalization, both of which appear to have exacerbated the patient’s psychosis. Additionally, the patient’s presentation to the hospital included decompensated psychosis which may have been precipitated partly by intranasal buspirone abuse from his outpatient prescriptions. However, the patient also presented with medication non-compliance, alcohol intoxication, and positive urine toxicology for amphetamines and tetrahydrocannabinol; therefore, it is likely that his initial psychosis was multifactorial. During the first hospital administration of buspirone, the patient had worsening of agitation and required multiple administrations of emergency antipsychotics in addition to his scheduled regimen. During the second administration, the patient’s paranoid delusions increased, and his oral intake could be used as a metric. Analyzing the average meal intake of the two periods of time immediately before and during the second buspirone administration, a student’s one-tailed *t*-test reveals a significant difference: t(53 degrees of freedom) = 4.83, *p* ≤ 0.0001. However, when comparing all hospitalization days of oral intake regardless of which buspirone window, there is only borderline significance that oral intake is worse on days that buspirone was administered: t(85 degrees of freedom) = 1.43, *p* = 0.08. The Naranjo (11) adverse reaction scale score was 5, indicating a probable cause between buspirone use and psychosis.

There are very limited reports between the use of buspirone and the worsening of psychosis (4–7, 12), and there are currently no conclusive reports on this reaction. Like earlier case reports published that suggest an association between psychosis and buspirone, our patient experienced worsening psychotic symptoms, including delusions, impaired thought process, hallucinations, and disorganized behavior. However, this cannot be concretely concluded as a direct result of buspirone alone due to the patient’s underlying diagnosis of schizoaffective disorder. Despite this, due to the chronological association of buspirone exposure with the patient’s exacerbated symptoms, it appears most likely that buspirone had a pathological influence. Diagnostically, the patient described is unable to meet the DSM-5-TR criteria for substance/medication-induced psychotic disorder due to his underlying schizoaffective disorder. Interestingly, the patient described does meet the DSM-5-TR diagnostic criteria for “other substance intoxication with perceptual disturbances” (ICD-10 code F19.122). If this adverse reaction is suspected in the future by other clinicians, it may be revealing to obtain serum levels of buspirone while psychosis is occurring, in addition to obtaining an objective and validated scale to measure psychosis, such as the Positive and Negative Syndrome Scale (PANSS).

Considering its primary mechanism of action, buspirone derives its neuropharmacological effects through 5-HT1A receptors. However, the drug also has been found to mediate dopamine neurotransmission. Initially developed as a neuroleptic, buspirone acts as an antagonist at presynaptic dopamine D2, D3, and D4 receptors, yet contrary to expected outcomes, it was unable to produce antipsychotic effects (13) and instead results in a substantial increase in dopaminergic metabolites such as homovanillic acid, norepinephrine (also from stimulation of the locus coeruleus) and its metabolite 3-methoxy-4-hydroxyphenylglycol (4, 9, 14). By inhibiting presynaptic rather than postsynaptic dopamine receptors, buspirone enhances dopamine neurotransmission by increasing the firing rate of dopamine neurons in the midbrain, similar to the mechanisms seen in the development of psychosis in schizophrenia (15). Therefore, it is possible that the worsening of psychosis while on buspirone may be due to its ability to manipulate the dopaminergic systems (16).

The route of administration may also enhance buspirone’s effects, considering that after first-pass metabolism, buspirone has approximately 4% oral bioavailability (17). Although the pharmacokinetics of oral buspirone are well known, there is currently limited data about intranasal bioavailability. In Swigart (18) described a case of buspirone abuse by nasal insufflation. A study in which buspirone hydrochloride nanovesicular *in situ* gel was administered intranasally was shown to prolongate its release and increase its bioavailability, allowing for a longer duration between doses (19). This may be due to the highly vascularized nature of the nasal mucosa, larger surface area, and porous endothelial membrane as well as bypassing the first pass effect as buspirone is metabolized by the hepatic enzyme cytochrome P450-3A4 (20, 21). In a study of a buspirone nasal drug delivery system, it was found that nasal administration led to 2.10 times increase in its bioavailability (19). This demonstrates that intranasal administration of buspirone leads to faster drug absorption by direct transport from the nasal mucosa to the brain (22). Intranasal administration of buspirone has been more commonly seen in prisons (23). It is reported that patients compared the sensation they receive from nasally inhaling buspirone to a euphoric feeling that can begin within minutes to an hour of receiving the drug (23).

## 4. Conclusion

Although buspirone is a generally safe anxiolytic azathioprine drug, it appears to have a rare adverse reaction of exacerbating psychosis in some patients. This may be associated with the intranasal administration of buspirone, resulting in increased bioavailability, which is more commonly seen in patients with stimulant use disorder and incarceration history.

## Data availability statement

The original contributions presented in this study are included in this article/supplementary material, further inquiries can be directed to the corresponding author.

## Ethics statement

The studies involving human participants were reviewed and approved by the Kern Medical Institutional Review Board. Written informed consent for participation was not required for this study in accordance with the national legislation and the institutional requirements.

## Author contributions

SA, RC, FH, KE, DW, and TT wrote the manuscript and formulated the analysis. All authors contributed to the article and approved the submitted version.
